# Suppurative necrotizing granulomatous lymphadenitis in adult-onset Still’s disease: a case report

**DOI:** 10.1186/1752-1947-6-354

**Published:** 2012-10-18

**Authors:** Stelios F Assimakopoulos, Vassilios Karamouzos, Christos Papakonstantinou, Vassiliki Zolota, Chryssoula Labropoulou-Karatza, Charalambos Gogos

**Affiliations:** 1 Department of Internal Medicine, University Hospital of Patras, Rion-Patras, 26504, Greece; 2 Department of Pathology, University Hospital of Patras, Rion-Patras, 26504, Greece

**Keywords:** Adult-onset Still’s disease, Granulomatous, Inflammatory, Lymphadenitis, Suppurative

## Abstract

**Introduction:**

Lymphadenopathy is found in about 65% of patients with adult-onset Still’s disease and is histologically characterized by an intense, paracortical immunoblastic hyperplasia. Adult-onset Still’s disease has not been previously described as an etiology of suppurative necrotizing granulomatous lymphadenitis.

**Case presentation:**

We describe a 27-year-old Greek man who manifested prolonged fever, abdominal pain, increased inflammatory markers, episodic skin rash and mesenteric lymphadenopathy histologically characterized by necrotizing granulomatous adenitis with central suppuration. Disease flares were characterized by systemic inflammatory response syndrome with immediate clinico-laboratory response to corticosteroids but the patient required prolonged administration of methylprednisolone at a dose of above 12mg/day for disease control. After an extensive diagnostic work-up, which ruled out any infectious, malignant, rheumatic or autoinflammatory disease the patient was diagnosed as having adult-onset Still’s disease. The patient is currently treated with 4mg of methylprednisolone, 100mg of anakinra daily and methotrexate 7.5mg for two consecutive days per week and exerts full disease remission for six months.

**Conclusion:**

To the best of our knowledge this is the first report of suppurative necrotizing granulomatous lymphadenitis attributed to adult-onset Still’s disease. This case indicates that the finding of a suppurative necrotizing granulomatous lymphadenitis should not deter the consideration of adult-onset Still’s disease as a potential diagnosis in a compatible clinical context; however, the exclusion of other diagnoses is a prerequisite.

## Introduction

Adult-onset Still’s disease (AOSD) is a systemic inflammatory disorder of unknown etiology and pathogenesis. There is no single diagnostic test for AOSD; rather, the diagnosis is based on clinical criteria, such as high daily fever, arthralgia, skin rash, lymphadenopathy, and hepatosplenomegaly, and exclusion of any infection, malignancy, or other rheumatic disorder known to mimic AOSD in its clinical features [1]. Lymphadenopathy is found in about 65% of AOSD patients and is histologically characterized by an intense, paracortical immunoblastic hyperplasia [1-3]. To the best of our knowledge, findings of suppurative necrotizing granulomatous lymphadenitis have not been previously described in AOSD.

The differential diagnosis of a necrotizing granulomatous lymphadenitis is wide, including: infectious diseases (bacterial, viral, fungal or parasitic); malignant disorders, mainly lymphoid malignancies; autoimmune disorders like systemic lupus erythematosus; autoinflammatory diseases; and idiopathic causes like Kikuchi’s disease and sarcoidosis [4-6]. The presence of variable degrees of suppuration although being suggestive of specific diagnoses like *Yersinia pseudotuberculosis* infection or tuberculosis (TB) does not preclude other potential causes of necrotizing granulomatous adenitis [4].

Here we describe a case of suppurative necrotizing granulomatous lymphadenitis attributed to AOSD after an extensive diagnostic work-up, which ruled out all the aforementioned etiologies.

## Case presentation

A 27-year-old Greek man, with an unremarkable past medical history except a reported penicillin allergy, was admitted to our department, referred from a regional hospital, for investigation of fever of three months’ duration associated with mesenteric lymphadenopathy, leukocytosis (25.000/mm^3^ with polymorphonuclear predominance) and increased erythrocyte sedimentation rate (ESR) over 100mm/h. The patient, who reported no close animal contact, had been previously hospitalized twice in the referral hospital complaining of fever associated with rigors and right lower guardant abdominal pain. The performed diagnostic investigation had only shown mesenteric lymphadenopathy and the patient was conservatively treated with intravenous antibiotic therapy consisting of ciprofloxacin and metronidazole with temporal improvement, followed by relapse after a short time.

On admission, the patient’s temperature was 39°C, heart rate was 90bpm, respiratory rate was 16 breaths per minute, blood pressure was 120/80mmHg and oxygen saturation in room air was 97%. His physical examination was unremarkable except for abdominal tenderness in his right lower guardant without rebound tenderness. No hepatomegaly, splenomegaly or peripheral lymphadenopathy was detected.

An initial laboratory evaluation showed a white blood cell count of 22.3 × 10^9^/L, with predominant neutrophils (80%), hematocrit 35%, hemoglobin 11.6g/dL (mean corpuscular volume = 82 and mean corpuscular hemoglobin = 26) and platelet count 51 × 10^9^/L. Prothrombin and partial thromboplastin times were normal and d-dimers were slightly increased at 0.72μg/ml. The blood biochemistries, including serum angiotensin-converting enzyme (ACE) and protein electrophoresis were all normal. Serum C-reactive protein was increased at 17.3mg/dL, ESR at 80mm/h, fibrinogen at 851mg/dL and ferritin at 663mg/dL. Thyroid function tests were normal. Urine analysis and 24-hour urinary calcium and protein excretion were normal. Electrocardiogram, chest X-ray, echocardiogram and arterial blood gas were also normal. A detailed ophthalmologic examination including slit-lamp eye examination, fundoscopy, Rose Bengal test and Schirmer’s test was unrevealing.

**Figure 1:**
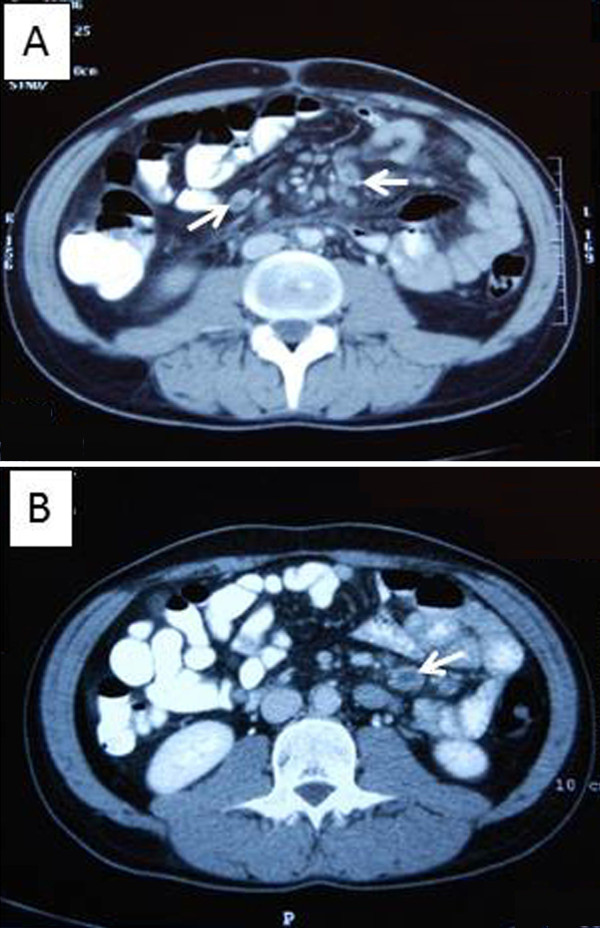
**Computed tomography scan of the patient’s abdomen (A and B):** A mesenteric lymphadenopathy of 2.7cm maximum diameter with a hypodense center was detected (white arrows)

An abdominal ultrasonography revealed enlarged mesenteric lymph nodes of 2.5cm diameter. Consequent thoracic and abdominal computed tomography (CT) scans confirmed the presence of mesenteric lymphadenopathy of 2.7cm maximum diameter with an hypodense center (Figure 1A, 1B), without evidence of bowel inflammation or hepatosplenomegaly, with normal vascular perfusion of abdominal organs, absence of mediastinal lymphadenopathy, ascites, pleural or pericardial effusions.

All sets of blood cultures (at least six), urine and stool cultures and examination for ova and parasites were negative. Antibodies for hepatitis A, B, C, coxsackie, Enteric Cytopathic Human Orphan virus, herpes simplex virus, Epstein–Barr virus, *cytomegalovirus*, human immunodeficiency virus, human T-lymphotropic virus-1 and virus-2, *Yersinia enterocolitica* and *Entamoeba histolytica*, *Bartonella henselae*, *Francisella tularensis*, *Leishmania donovani*, *Coxiella burnetii* and *Rickettsia conorii* were negative. Wright and rapid plasma reagin tests were also negative, whereas the tuberculin skin test was positive (15mm).

The patient underwent upper and lower gastrointestinal tract endoscopies, enteroclysis and capsule endoscopy without evidence of inflammatory bowel disease, infective colitis or celiac disease. Examination of gastric fluid with Ziehl–Neelsen stain detected no acid-fast bacteria; a polymerase chain reaction (PCR) as well as culture for mycobacterium TB were negative. Small bowel (jejunal and ileal) and colonic biopsies presented findings of non-specific inflammatory reaction, the architecture of intestinal villi was preserved and mucosal periodic acid-Schiff stain for detection of *Tropheryma whipplei* was negative.

A full immunologic screening with rheumatoid factor, antinuclear antibodies, antibodies to double-stranded DNA, anti-Sm, anti-Ro/SSA, anti-La/SSB, anti-RNP, anti-Jo-1, anti-Scl-70, anti-histones, anti-mitochondrial antibodies, anti-smooth muscle antibodies, cytoplasmic-antineutrophil cytoplasmic antibody (ANCA), perinuclear-ANCA, anti-transglutaminase, anti-cardiolipin, and lupus anticoagulant was negative. Serum complement and levels of immunoglobulins (Igs, IgA, IgG, IgM, IgE, IgD) were normal. The results of genetic testing for mutation of the familial Mediterranean fever gene (Μ694V, V726A, M694I, M680I, and E148Q) were negative. In addition, the results of a full tumor marker profile were also normal.

**Figure 2:**
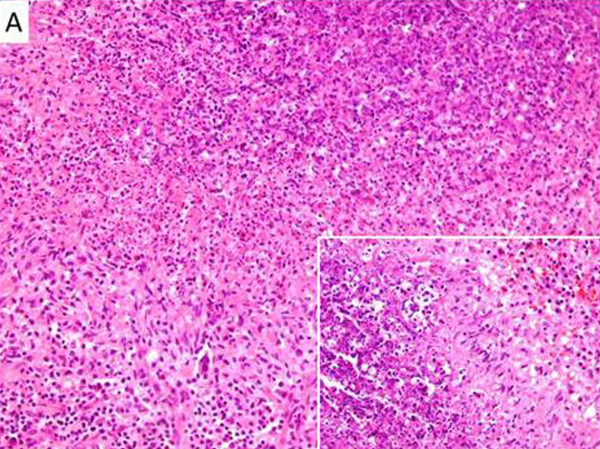
**Mesenteric lymph node biopsy from the presented patient:** formation of histiocytic granulomas with central suppurative necrosis (hematoxylin and eosin stain, A: ×100, insert ×200)

The patient underwent laparotomy with mesenteric lymph node excision for microbiological and histological examination. Histology revealed a granulomatous lymphadenitis with central suppurative necrosis (Figure 2A). Gram, Giemsa, Ziehl–Neelsen and Grocott methenamine silver stains for detection of common bacteria, mycobacteria and fungi were all negative. Immunohistochemical studies for lymphoproliferative disease were unrevealing. In addition, the patient’s lymph node was examined with a PCR and cultures for mycobacterium TB, atypical mycobacteria (*Mycobacterium africanum* I and/or II, *M. microti*, *M. carnetti*, *M. bovis*, and *M. avium* complex), fungi and *Tropheryma whipplei* without detection of any pathogen. A gastrocnemius muscle biopsy was also performed without identification of sarcoid granulomata.

The hematological work-up in the investigation of the patient included microscopic examination of peripheral blood film which showed no evidence of lymphoproliferative disorders. A bone marrow biopsy and immunophenotypic analysis revealed no pathologic findings. A peripheral blood immunophenotypic analysis revealed lymphopenia due to simultaneous decrease of B-, T- and NK-cells. Serum beta-2-microglobulin levels were within normal limits.

After an extensive diagnostic work-up no definite diagnosis was available for our patient. During hospitalization the patient was empirically treated with combined antimicrobial therapy that consisted of ciprofloxacin and metronidazole without response, while episodic disease flares characterized by a systemic inflammatory response syndrome (SIRS) and spikes of increased markers of inflammation were recorded. Sporadically, fever spikes were associated with a transient maculopapular skin rash in his upper extremities. Of note, the patient experienced a dramatic clinical improvement after administration of corticosteroids for a short time; the corticosteroids were administered due to his history of allergy in order to administer intravenous contrast media for CT performance. This temporal improvement was shortly followed by disease relapse. Based on the therapeutic criterion of response to corticosteroids and taking into consideration the significantly positive tuberculin skin test in conjunction with the histology of the mesenteric lymph node, which showed a granulomatous necrotizing lymphadenitis with central suppuration, we decided to administer a combination empiric therapy. The therapy consisted of corticosteroids (methylprednisolone 16mg) and anti-TB drugs (isoniazide 150mg/d, pyrazinamide 30mg/kg, ethambutol 20mg/kg and moxifloxacin 400mg/d). Rifampicin 300mg/d, which was included in our initial scheme, was later discontinued due to induction of rifampicin-associated pancreatitis. He received the anti-TB therapy for one year and discontinued corticosteroids after a very slow tapering at 11 months. During this period the patient was asymptomatic with normal values of inflammatory markers and he had a total resolution of mesenteric lymphadenopathy at abdominal CT re-examination on completion of the anti-TB therapy. Unfortunately, 20 days after stopping the corticosteroids the disease relapsed, and the patient had fever, abdominal pain, increased markers of inflammation and reappearance of mesenteric lymphadenopathy on abdominal MRI. The patient’s symptoms were controlled with 16mg methylprednisolone; however, while attempting to taper the methylprednisolone to 12mg after about two months of treatment, his symptoms relapsed forcing us to reinstitute a higher corticosteroid dose. Our second effort to gradually reduce the corticosteroid dose, after control of the patient’s symptoms and total remission of mesenteric lymphadenopathy on abdominal MRI, led to disease relapse when the dose of methylprednisolone was reduced to 12mg/day. Disease relapse was controlled by increasing methylprednisolone dose to 16mg, while additional immunomodulatory drugs (anakinra and methotrexate) were added in order to prevent disease relapse during methylprednisolone tapering. The patient is currently treated with 4mg of methylprednisolone, 100mg of anakinra daily and methotrexate 7.5mg for two consecutive days per week and has experienced full disease remission for six months.

## Discussion

Here we describe a previously healthy young man who presented with prolonged fever, abdominal pain, increased inflammatory markers, episodic skin rash and mesenteric lymphadenopathy histologically characterized by necrotizing granulomatous adenitis with central suppuration. Disease flares were characterized by SIRS with immediate clinico-laboratory response to corticosteroids but the patient required prolonged administration of methylprednisolone at a dose of above 12mg/day for disease control.

In Table 1 we present an extensive differential diagnosis of granulomatous necrotizing and suppurative lymphadenitis [4,5,7]. As shown in Table 1, histological overlapping of the formation, necrosis and suppuration of granulomas, in variable degrees, may exist in diverse lymphadenopathies. Therefore, when a suppurative necrotizing granulomatous lymphadenitis cannot be associated with any of its typical causes, our diagnostic consideration should encompass all the potential causes of granulomatous and necrotizing lymphadenitis.

**Table 1:** Causes of granulomatous and/or necrotizing lymphadenitis, with or without suppuration

	**Granulomas**	**Necrosis**	**Suppuration**
A. **Infections**			
***Viruses***	–	+	–
(EBV, CMV, HBV, HCV, Herpes simplex, Adenovirus, HIV)			
***Bacteria***			
*Yersinia pseudotuberculosis*	+	+	+
*Bartonella*	+	+	+
*Tropheryma whipplei*	+	–	–
*Francisella tularensis*	+	+	+
*Brucella*	+	+	+
***Spirochaetes***			
*Treponema pallidum*	+	+	+
***Chlamydia***			
Lymphogranuloma venereum	+	+	+
***Rickettsia***			
*Coxiella burnetii*	+	–	–
***Mycobacteria***			
*Mycobacterium tuberculosis*	+	+	+
Atypical mycobacterial infection	+	+	+
*Mycobacterium leprae*	+	+	+
***Parasites***			
Toxoplasmosis	+	±	+
Leishmaniasis	+	–	–
***Fungi***			
Histoplasmosis	+	+	+
Aspergillosis	+	–	–
Coccidioidomycosis	+	+	+
Cryptococcosis	+	+	+
B. **Neoplastic diseases**			
Hodgkin disease	+	+	±
Non-Hodgkin disease	±	+	±
Metastatic carcinoma	+	+	±
Langerhans cell histiocytosis	+	–	–
Seminoma	+	–	–
Dysgerminoma	+	–	–
C. **Autoimmune diseases**			
Systemic lupus erythematosus	–	+	–
Granulomatosis with polyangiitis	+	+	+
Churg–Strauss syndrome	+	+	–
Celiac disease	+	–	–
Crohn’s disease	+	–	–
Primary biliary cirrhosis	+	+	–
Kawasaki disease	–	+	+
D. **Idiopathic diseases**			
Sarcoidosis	+	–	–
Necrotic sarcoid granulomatosis	+	+	–
Kikuchi-Fujimoto disease	+	+	–
E. **Autoinflammatory diseases**			
Familial Mediterranean fever	–	+	–
Hyperimmunoglobulinemia-D syndrome	no data	no data	no data
PFAPA syndrome	–	±	±

Note: ± indicates the potential existence of the corresponding histological feature, which is however non-typical for this disease.

CMV, *Cytomegalovirus*; EBV, Epstein–Barr virus; HBV, Hepatitis B; HCV, Hepatitis C; HIV, Human immunodeficiency virus; PFAPA, Periodic fever, aphthous stomatitis, pharyngitis and adenitis.

In our patient, no infectious etiology could be detected by serological, microbiological, histological and molecular methods. Whipple’s disease, which was a serious diagnostic consideration, was thoroughly investigated and excluded. Lymph node histology in conjunction with positive Mantoux reaction, compatible clinical picture, and absence of an alternative confirmed diagnosis led to institution of empiric anti-TB therapy. Disease relapse after completion of one year’s anti-TB therapy was the strongest criterion for exclusion of tuberculous lymphadenitis.

Lymphoid malignancies, either non-Hodgkin or Hodgkin disease, were excluded based on lymph node histologic and immunohistochemical studies, bone marrow aspiration, biopsy and immunophenotypic analysis, and peripheral blood smear examination and immunophenotypic analysis.

Crohn’s disease was another important diagnostic possibility. Normal upper and lower gastrointestinal tract endoscopies, enteroclysis, capsule endoscopy and small and large intestinal biopsies excluded this diagnosis.

Sarcoidosis was also considered to be a diagnostic possibility in our patient. The typical histological feature of sarcoidosis is the formation of non-necrotizing granulomata [8]; however, the existence of necrotizing sarcoid granulomatosis has also been described since 1973 [9]. Normal serum ACE levels, absence of hypercalciuria, normal pulmonary function tests, slit-lamp eye examination, CD4 to CD8 ratio in bronchoalveolar lavage, and no detection of sarcoid granulomata in gastrocnemius muscle biopsy [10], made this diagnosis unlikely.

Regarding autoimmune diseases, systemic lupus erythematosus was excluded by the absence of autoantibodies. The possibility of granulomatosis with polyangiitis and Churg–Strauss syndrome was ruled out based mainly on clinical criteria combined with negative ANCAs. Kawasaki disease, which occurs rarely in adults, was excluded by incompatible clinical course because it is typically a self-limited condition, while its associated necrotizing lymphadenitis is typically non-granulomatous [11].

Kikuchi-Fujimoto disease was excluded based on the prolonged and relapsing clinical course of our patient and the histological features of affected lymph nodes with significant infiltration by neutrophils [12]. Among autoinflammatory diseases, the familial Mediterranean fever was excluded by appropriate genetic testing, the hyper-IgD syndrome by normal serum IgD and IgA levels and periodic fever with aphthous stomatitis, pharyngitis, and adenitis syndrome by clinical criteria [13].

After the aforementioned extensive diagnostic work-up and ruling out all potential alternative diagnostic considerations, AOSD, an inflammatory disorder which could be expressed with daily fevers, increased inflammatory markers and lymphadenopathy, in the absence of positive autoantibodies, came into play as an important diagnostic consideration. Disease presentation in the patient was atypical for AOSD because it lacked common features such as arthralgias, sore throat, abnormal liver function tests and significant serum hyperferritinemia [14]. However, diagnosis of AOSD was based on fulfillment of the commonly used high sensitive Yamaguchi criteria (93.5% sensitivity) and exclusion of any other diagnostic consideration including any infectious, malignant, or rheumatic disorder known to mimic AOSD in its clinical features (Table 2) [1,15]. Although a lymph node biopsy is not necessary for the diagnosis of AOSD it is often performed to rule out other potential diagnostic thoughts, like lymphomas. Lymphadenopathy in AOSD patients is histologically characterized by an intense, paracortical immunoblastic hyperplasia, whereas the finding of a suppurative necrotizing granulomatous lymphadenitis has not been previously described [1-3].

**Table 2:** Accordance of the presented patient with the Yamaguchi criteria for the diagnosis of adult-onset Still’s disease

**Yamaguchi criteria (require the presence of five features, with at least two being major diagnostic criteria):**	**Patient’s characteristics**
Major Yamaguchi criteria:	
1. Fever of at least 39°C lasting at least one week.	+
2. Arthralgias or arthritis lasting two weeks or longer.	–
3. Typical rash (maculopapular, nonpruritic) during febrile episodes.	+
4. Leukocytosis (10,000/μL or greater), with at least 80% granulocytes.	+
Minor Yamaguchi criteria:	
1. Sore throat	–
2. Lymphadenopathy	+
3. Hepatomegaly or splenomegaly	–
4. Abnormal liver function studies	–
5. Negative antinuclear antibodies and rheumatoid factor.	+
Exclusions	
1. Infection, especially sepsis and infectious mononucleosis	✓
2. Malignancies, especially lymphomas	✓
3. Rheumatic diseases known to mimic adult Still’s disease	✓

## Conclusion

To the best of our knowledge this is the first report of suppurative necrotizing granulomatous lymphadenitis attributed to AOSD. The presented case demonstrates that the finding of a suppurative necrotizing granulomatous lymphadenitis should not deter the consideration of AOSD as a potential diagnosis when the clinical features are compatible; however, exclusion of a wide range of infections, malignancies and other rheumatologic conditions should always be performed.

## Consent

Written informed consent was obtained from the patient for publication of this case report and accompanying images. A copy of the written consent is available for review by the Editor-in-Chief of this journal.

## Abbreviations

ACE: angiotensin-converting enzyme; ANCA: antineutrophil cytoplasmic antibody; AOSD: Adult-onset Still’s disease; CT: computed tomography; ESR: erythrocyte sedimentation rate; Ig: immunoglobulin, PCR, polymerase chain reaction; SIRS: systemic inflammatory response syndrome; TB: tuberculosis.

## Competing interests

The authors declare that they have no competing interests.

## Authors’ contributions

SFA wrote this case report; SFA, VK and CP, were the patient’s doctors; VZ performed the histological diagnosis; CL-K and CG critically revised the manuscript. All authors have read and approved the final version of this manuscript.
